# Common Variants in *CRP* and *LEPR* Influence High Sensitivity C-Reactive Protein Levels in North Indians

**DOI:** 10.1371/journal.pone.0024645

**Published:** 2011-09-08

**Authors:** Anubha Mahajan, Rubina Tabassum, Sreenivas Chavali, Om Prakash Dwivedi, Ganesh Chauhan, Saurabh Ghosh, Nikhil Tandon, Dwaipayan Bharadwaj

**Affiliations:** 1 Genomics and Molecular Medicine Unit, CSIR-Institute of Genomics and Integrative Biology, Delhi, India; 2 Human Genetics Unit, Indian Statistical Institute, Kolkata, India; 3 Department of Endocrinology, All India Institute of Medical Sciences, New Delhi, India; University of Bristol, United Kingdom

## Abstract

**Background:**

High sensitivity C-reactive protein (hsCRP) levels are shown to be influenced by genetic variants in Europeans; however, little is explored in Indian population.

**Methods:**

Herein, we comprehensively evaluated association of all previously reported genetic determinants of hsCRP levels, including 18 *cis* (proximal to *CRP* gene) and 73 *trans*-acting (distal to *CRP* gene) variants in 4,200 North Indians of Indo-European ethnicity. First, we evaluated association of 91 variants from 12 candidate loci with hsCRP levels in 2,115 North Indians (1,042 non-diabetic subjects and 1,073 patients with type 2 diabetes). Then, *cis* and *trans*-acting variants contributing maximally to hsCRP level variation were further replicated in an independent 2,085 North Indians (1,047 patients with type 2 diabetes and 1,038 non-diabetic subjects).

**Results:**

We found association of 12 variants from *CRP*, *LEPR*, *IL1A*, *IL6*, and *IL6R* with hsCRP levels in non-diabetic subjects. However, only rs3093059-*CRP* [β = 0.33, *P* = 9.6×10^−5^] and the haplotype harboring rs3093059 risk allele [β = 0.32 µg/mL, *P* = 1.4×10^−4^/*P_perm_* = 9.0×10^−4^] retained significance after correcting for multiple testing. The *cis*-acting variant rs3093059-*CRP* had maximum contribution to the variance in hsCRP levels (1.14%). Among, *trans*-acting variants, rs1892534-*LEPR* was observed to contribute maximally to hsCRP level variance (0.59%). Associations of rs3093059-*CRP* and rs1892534-*LEPR* were confirmed by replication and attained higher significance after meta-analysis [β*_meta_* = 0.26/0.22; *P_meta_* = 4.3×10^−7^/7.4×10^−3^ and β*_meta_* = −0.15/−0.12; *P_meta_* = 2.0×10^−6^/1.6×10^−6^ for rs3093059 and rs1892534, respectively in non-diabetic subjects and all subjects taken together].

**Conclusion:**

In conclusion, we identified rs3093059 in *CRP* and rs1892534 in *LEPR* as major *cis* and *trans*-acting contributor respectively, to the variance in hsCRP levels in North Indian population.

## Introduction

High sensitivity C-reactive protein (hsCRP), is a key component of non-specific host defense in humans. Elevated basal hsCRP levels have been found in low-grade systemic inflammation and are used as a marker of risk for various pathological conditions including type 2 diabetes (T2D) [1], [2]. These levels are genetically determined with ∼30–50% heritability [3].

Both *cis*- (proximal to *CRP* gene) and *trans*-acting (distal to *CRP* gene) genetic variants have been identified to contribute to hsCRP levels in different ethnic cohorts [4], [5], [6], [7], [8], [9], [10]. Recent genome-wide association studies (GWAS) have confirmed the involvement of several previously identified variants and additionally recognized new variants, providing important insights into hsCRP level regulation [11], [12]. However, very little is explored in this regard in Indian population, which is a high risk group for T2D. Elevated levels of hsCRP were found to be associated with increased risk of T2D in North Indian population in our previous study [13]. Moreover, our recent analysis of *TNF-LTA* locus identified a variant (rs2229094) influencing hsCRP levels in this population [14]. Thus, a more thorough examination of genetic determinants of hsCRP levels in this population is warranted.

Herein, we comprehensively evaluated association of all previously reported genetic determinants of hsCRP levels in North Indians.

## Materials and Methods

### Ethics Statement

The study was carried out in accordance with the principles of Helsinki Declaration and was approved by Human Ethics Committee of Institute of Genomics and Integrative Biology (CSIR) and All India Institute of Medical Sciences Research Ethics Committee. Informed written consent was obtained from all the participants.

### Study population

The initial investigation included 2,115 unrelated Indo-Europeans (1,073 T2D patients and 1,042 non-diabetic subjects) from North India residing in and around Delhi. Details of subject recruitment have been described previously [13], [14], [15]. An independent set of 2,085 samples (1,047 T2D patients and 1,038 non-diabetic subjects), recruited similar to that in the initial study, was used for further replication. Plasma hsCRP levels were measured using ELISA kit (Biocheck Inc., CA, USA) and Cobas Integra 400 Plus (Roche Diagnostics, GmbH, Mannheim, Germany).

### Variant selection and genotyping

We selected all the index variants shown to be associated with hsCRP levels till July 2009 or their proxies. Further, to capture other variants in the selected genes, we also included additional common variants that were prioritized based on minor allele frequency (MAF >0.05) in at least two HapMap populations, tagging and linkage disequilibrium (LD) pattern information, and spacing between the variants to cover the gene. The selected variants comprised of 18 *cis* variants from *CRP* and 73 from 11 *trans*-acting loci (*APOE*, *LEPR*, *IL1A*, *IL1B*, *IL6*, *IL6R*, *FAM13C1*, *GCKR*, *SLC1A3*, *HNF1A*, and a gene-desert region of chromosome 12q23.2) (Table S1).

Genotyping of 47 variants was done using Illumina GoldenGate assay [16]. Samples (n = 90) with a poor call rate (<90%) and variants (n = 3) failing quality control were excluded from the analysis. Average call rate for successful assays was 99.6% with >99.9% concordance in 7.2% duplicates. The remaining 44 variants and one that failed Illumina assay were genotyped using iPLEX (Sequenom, San Diego, USA). The average genotyping success rate of the quality control passed variants (n = 41) was 97.7%, with 99.9% consensus in 4% duplicates. Four rare variants (MAF<0.05) and three that deviated from Hardy-Weinberg equilibrium (*P*<5.9×10^−4^; 0.05/85) were excluded, and thus 78 variants were considered for association analyses.

Further genotyping of two variants was done in an independent sample set on iPLEX platform for replication. Average genotyping success rate for these was 98.3% with 99.9% concordance in 5% duplicates.

### Statistical analysis

Linear-regression was performed assuming an additive model to determine association of variants with hsCRP levels. Samples with hsCRP level >10 µg/mL were excluded from the analysis. hsCRP concentrations were natural logarithm transformed to ensure a normal distribution. To assess independent contribution of each variant, stepwise multivariate linear regression models with age, sex, and body mass index (BMI) as covariates were constructed. Variants with a *P*<0.05 were retained in the forward stepwise-selection models. Analyses were first carried out in non-diabetic subjects not taking lipid-lowering drugs, adjusting for age, sex, BMI, and smoking/tobacco chewing and then in T2D patients and all subjects, additionally adjusting for diseases status and medication as appropriate.

Bonferroni correction was used to account for multiple testing and a *P*<8.6×10^−4^ was considered significant after correcting for number of independent variants α = 0.05/58). Pairwise linkage disequilibrium (LD) between the SNPs for all the genes was determined using Haploview 4.0 (Figure S1). Association of haplotypes with frequency >0.1 was examined through sliding window approach using PLINK by adjusting for age, sex, BMI, smoking/tobacco chewing, diseases status, and medication, as appropriate, based on 10,000 permutations. Fixed and random effect meta-analysis was performed for the two replicated variants, in non-diabetic subjects, T2D patients and all subjects.

Statistical power of the study was evaluated using QUANTO version 1.2 [17]. Our study had 78% and 92% power to detect association for a variant with MAF = 0.2 and effect size of 0.3 µg/mL for additive model for non-diabetic subjects and for all subjects analyzed together respectively. Statistical analyses were performed using SPSS version 17.0 (SPSS, Chicago, IL) and PLINK [18].

## Results

A brief description of study populations is presented in Table S2.

### *Cis*-acting effects

*CRP* variants rs3093059 and rs3093077, that were in strong LD (r^2^ = 0.89), showed strongest associations with hsCRP in non-diabetic subjects [β = 0.32 µg/mL, *P* = 1.6×10^−4^ and β = 0.26, *P* = 1.3×10^−3^ respectively; Table 1]. Nominal associations were observed for variants rs3116654, rs4131568, and rs1205. A total of 2.42% of the residual variation in hsCRP could be explained by variants rs3093059, rs3116654, and rs4131568. Variant rs3093059 contributed maximally (1.14%) to hsCRP level variance and also retained significant association with hsCRP levels after accounting for multiple testing. The haplotype GATCTTCG encompassing rs3093059 risk allele also showed association with hsCRP levels [β = 0.32, *P* = 1.4×10^−4^/*P*_perm_ = 9.0×10^−4^; Table S3]. We observed similar results when separate analyses for T2D patients and all the subjects were performed (Table 1). Further, we tested association of haplotypes of different lengths to detect a signal that could not be observed by single marker analysis. The β and *P* values for haplotype associations were found to be similar as the individual associated SNPs (Figure S2). We did not find any haplotype that could detect region or signal in addition to single marker analysis.

**Table 1:** Variants associated with hsCRP levels.

Gene	Polymorphism	MAF*	Median hsCRP level (µg/mL)*			Non-diabetic subjects (n = 910)		T2D subjects (n = 790)		All subjects (n = 1,700)	
			11	12	22	β‡	P value‡	β∥	P value∥	β^§^	P value^§^
*CRP*	rs2592887	0.34	1.12	1.27	0.96	−0.05	0.37	−0.24	**4.9×10^−4^**	−0.13	4.3×10^−3^
	rs2794520	0.29	1.17	1.18	0.96	−0.11	0.06	−0.20	4.2×10^−3^	−0.15	1.1×10^−3^
	rs3093077	0.13	1.09	1.53	1.43	0.26	1.3×10^−3^	0.26	0.01	0.26	**3.8×10^−5^**
	rs1205	0.29	1.17	1.19	0.97	−0.12	0.05	−0.21	1.9×10^−3^	−0.16	**5.3×10^−4^**
	rs3091244 †	0.34	1.10	1.16	1.41	0.08	0.14	0.13	0.06	0.10	0.03
	rs3093059	0.12	1.09	1.61	1.32	0.32	**1.6×10^−4^**	0.29	7.1×10^−3^	0.31	**2.8×10^−6^**
	rs3116654	0.10	1.17	1.19	0.30	−0.23	0.02	−0.07	0.59	−0.18	0.02
	rs4131568	0.27	1.25	1.15	1.11	−0.13	0.03	−0.06	0.40	−0.11	0.02
*LEPR*	rs1805096	0.48	1.35	1.17	1.13	−0.13	0.02	−0.11	0.06	−0.12	3.2×10^−3^
	rs1892534	0.48	1.36	1.15	1.13	−0.16	5.5×10^−3^	−0.12	0.07	−0.14	1.3×10^−3^
	rs12753193	0.48	1.42	1.14	1.13	−0.15	8.3×10^−3^	−0.10	0.12	−0.13	3.1×10^−3^
*IL6R*	rs4845371	0.50	1.20	1.26	1.10	−0.11	0.06	−0.09	0.16	−0.10	0.02
	rs6667434	0.50	1.20	1.26	1.10	−0.11	0.06	−0.09	0.16	−0.10	0.02
	rs7529229	0.31	1.26	1.17	1.08	−0.14	0.02	−0.03	0.69	−0.10	0.04
	rs4129267	0.29	1.25	1.12	1.10	−0.13	0.04	−0.02	0.79	−0.09	0.08
	rs4845622	0.32	1.24	1.13	1.08	−0.12	0.04	−0.03	0.75	−0.08	0.07
*IL1A*	rs1800587	0.33	1.20	1.11	1.45	0.12	0.04	−0.04	0.57	0.06	0.20
*IL6*	rs1800797	0.17	1.15	1.21	1.53	0.06	0.43	0.25	3.8×10^−3^	0.14	0.01
	rs2069827	0.05	1.17	1.41	1.36	0.02	0.83	0.31	0.03	0.13	0.14
	rs2069845	0.24	1.18	1.11	1.43	−0.005	0.94	0.15	0.05	0.06	0.24
	rs1800796	0.28	1.32	1.08	1.23	−0.14	0.02	−0.15	0.02	−0.14	1.5×10^−3^
*APOE*	rs157580	0.46	1.08	1.15	1.44	0.08	0.12	0.18	5.1×10^−3^	0.12	3.7×10^−3^
	rs2075650	0.11	1.17	1.10	1.62	−0.11	0.20	−0.24	9.8×10^−3^	−0.17	7.8×10^−3^
	rs769449	0.07	1.18	1.03	1.33	−0.19	0.09	−0.39	1.7×10^−3^	−0.27	1.2×10^−3^
*GCKR*	rs13013484	0.33	1.23	1.10	1.26	−0.007	0.91	−0.19	5.7×10^−3^	−0.07	0.09
*IL1B*	rs1143642	0.11	1.16	1.15	1.49	0.01	0.87	0.24	0.02	0.11	0.10

MAF: Minor allele frequency; 11: Homozygous major; 12: Heterozygous; 22: Homozygous minor; β: Beta.

*Analyzed in only non-diabetic subjects.

† Tri-allelic variant. The two less-common alleles (T and A) were pooled.

‡ *P* value and β calculated after adjusting for age, sex, BMI, and smoking/tobacco chewing.

∥ *P* value and β calculated after adjusting for age, sex, BMI, smoking/tobacco chewing, and medication^§^ *P* value and β calculated after adjusting for age, sex, BMI, smoking/tobacco chewing, medication, and diseases status.

*P* values retaining significance after correcting for multiple testing are given in bold.

### *Trans*-acting effects

Three *LEPR*, two *IL6R*, one *IL6*, and one *IL1A* variants showed association with hsCRP levels (*P*<0.05; Table 1). All three *LEPR* and two *IL6R* variants (rs4129267, and rs4845622) were in strong LD (r^2^ = 0.91–0.96 and 0.96 respectively). The most significant association was detected for *LEPR*-rs1892534 [β = −0.16, *P* = 5.5×10^−3^], however, none of the associations retained significance after accounting for multiple testing. These loci accounted for a total of 1.63% hsCRP level variance, with 0.59%, 0.56%, and 0.48% variance attributed to rs1892534-*LEPR*, rs1800796-*IL6*, and rs4129267-*IL6R* respectively. *LEPR*, *IL6* and *IL6R* also showed association with hsCRP levels when all subjects were analyzed together. The most significant association was observed for rs769449-*APOE* [β = −0.27, *P* = 1.2×10^−3^]. However, none of these retained significance after correcting for multiple testing. Similar results were obtained when analyses were performed separately in T2D patients (Table 1). The haplotype GGGCCA-*IL6* was associated with hsCRP levels when all the subjects were analyzed together [β = −0.16, *P* = 3.9×10^−4^/*P*_perm_ = 1.2×10^−3^]. We did not find any haplotype that could detect region or signal in addition to single marker analysis (Figure S2).

### Follow up and Meta-analysis

The rs3093059-*CRP* and rs1892534-*LEPR*, strongest associated *cis* and *trans*-acting variants respectively, were successfully replicated in an independent sample of North Indians (Table 2). Meta-analysis further strengthened the associations in non-diabetic subjects [β*_meta_* = 0.26, *P_meta_* = 4.3×10^−7^ and β*_meta_* = −0.15, *P_meta_* = 2.0×10^−6^ for rs3093059 and rs1892534, respectively, for random effect]. No heterogeneity was detected between two study populations (I^2^ = 0). Similarly, significant associations were observed when all subjects were analyzed together, however only nominal association of rs1892534-*LEPR* was observed when analysis was performed separately in T2D patients (Table 2).

**Table 2:** Association of rs3093059-*CRP* and rs1892534-*LEPR* with hsCRP levels.

		Initial phase		Follow up		Meta-analysis				
		Non-diabetic: 910		Non-diabetic: 961		Non-diabetic: 1,871				
Polymorphism (Gene)	Subjects	T2D: 790		T2D: 903		T2D: 1693				
		All:1,700		All:1,864		All: 3,564				
						Random effect		Fixed effect		
		β	P	β	P	β_r_	P_r_	β_f_	P_f_	I^2^
rs3093059	Non-diabetic	0.32	1.6×10^−4^	0.22	5.4×10^−4^	0.26	4.3×10^−7^	0.26	4.3×10^−7^	0
(*CRP*)	Diabetics	0.29	7.1×10^−3^	0.01	0.90	0.14	0.31	0.12	0.08	0.76
	All	0.31	2.8×10^−6^	0.17	4.5×10^−3^	0.22	7.4×10^−3^	0.21	3.0×10^−7^	0.75
rs1892534	Non-diabetic	−0.16	5.5×10^−3^	−0.14	1.2×10^−4^	−0.15	2.0×10^−6^	−0.15	2.0×10^−6^	0
(*LEPR*)	Diabetics	−0.12	0.07	−0.07	0.13	−0.09	0.02	−0.09	0.02	0
	All	−0.14	1.3×10^−3^	−0.10	3.1×10^−4^	−0.12	1.6×10^−6^	−0.12	1.6×10^−6^	0

β_r_: Beta for random-effects; β_f_ Beta for fixed-effects; *P_r_*: *P* value for random-effects; *P_f_*: *P* value for fixed-effects; I^2^: Heterogeneity index (0–100).

## Discussion

Recent GWAS and other previous candidate gene based investigations have demonstrated role of c*is-*acting variants of *CRP* and *trans-*acting variants from genes including *LEPR*, *IL6R*, *IL6* etc in regulation of hsCRP levels. Our study demonstrated strong association of a *cis*-acting variant rs3093059-*CRP* and a *trans*-acting variant rs1892534-*LEPR* with hsCRP levels in North Indians. These two variants contributed maximally to the variance in hsCRP levels.

Several studies have shown strong association of variants at *CRP* locus with plasma levels of hsCRP [7], [19]–[22]. Our study also demonstrates *cis* acting variants to greatly influence plasma hsCRP levels. Among them, rs3093059 showed strongest association with hsCRP levels that was confirmed in an independent study population. The results in the combined analysis of T2D patients and non-diabetic subjects were comparable to that seen in non-diabetic subjects alone. Our observations are consistent with previous reports demonstrating that both *CRP* variants and haplotypes are associated with hsCRP levels [7], [19].

Among the *trans* acting variants, strongest association was observed at rs1892534 of *LEPR.* Leptin receptor signaling has been linked to appetite control, weight regulation, glucose homeostasis, vascular risk, and hsCRP levels [8], [11]. Previous reports provided evidence for the association of rs1892534 with hsCRP levels in European populations [8], [11]. Thus, our results support the hypothesis that leptin receptor influence inflammatory traits through genetic modulation of hsCRP levels.

We found nominal association of other *trans-*acting loci namely *IL1A*, *IL6*, *IL6R*, and *APOE* with hsCRP levels in non-diabetic North Indians. All these variants contributed to ∼2% of hsCRP level variance. The analysis of all subjects taken together essentially substantiated the results seen in non-diabetic subjects. *IL6R* and *APOE* variants were shown to be associated with hsCRP levels in apparently healthy Caucasian women in an agnostic approach [11]. This is perhaps the first study to replicate the association of rs1800587-*IL1A* variant [5], though it could not attain the statistical significance after multiple testing corrections. The effects of all associations were directionally consistent and the effect sizes were almost comparable to earlier studies [5], [8], [11]. Hence, our study validates the observations in European populations, suggesting involvement of overlapping genetic mechanisms in regulating hsCRP levels in Europeans and North Indians.

Though we replicated the association of *CRP*, *LEPR*, *IL1A*, *IL6*, *IL6R*, and *APOE*, we could not detect association of the most widely implicated tri-allelic *CRP* variant rs3091244. The tri-allelic variant rs3091244 has been repeatedly shown to be associated with plasma hsCRP levels and an *in vitro* study suggested its functional role in transcription factor binding [20]. There was no difference in allele frequencies between Caucasians and North Indians at this locus but we observed differences in LD pattern around this variant in the *CRP* gene between the two populations (Figure S1). A recent GWAS study for hsCRP plasma levels also indicated the differences in genetic background in *CRP* between different ethnic groups [21]. Thus the ethnic differences in genetic architecture at this locus could be the reason for difference in association results.

Moreover recent studies have discovered *GCKR* as a novel hsCRP modulating candidate gene and have provided confirmatory evidence for the association of *HNF1A* [9], [11]. However, variants from both these loci were not associated in our study. Allele frequencies at these loci differed significantly between the Caucasians and North Indians and could have potentially limited the power to detect association, if any. Differences in LD patterns between the populations could potentially lead to differences in results but the comparison of LD around these genes showed similar patterns in Indians and Europeans (Figure S1) and thus do not account for the observed differences in association results.

Limited statistical power and population stratification might also lead to the disparity in the association results. There is a likelihood of false negative observations for variants with small effect sizes, as our study is sufficiently powered to capture only large effects (>0.25 µg/mL) of the common variants with frequencies more than 0.20. Our study is, however, unlikely to be influenced by population stratification, because the study population is highly homogeneous, as previously demonstrated [15], [23]. We have included only North Indian subjects belonging to Indo-European ethnicity from Delhi and surrounding regions that form a homogenous cluster as reported by the Indian Genome Variation Consortium [23].

Further, we would like to mention that although we attempted to cover entire gene and investigate all the reported associated variants, we could not type few of them due to assay failures. Moreover, the ascertainment procedure of the subjects that recruited subjects based on the presence or absence of T2D could potentially affect the estimation of effect sizes. We could not determine the population level effect sizes that could have provided added information from the study. However, with the major focus of the study to determine the genetic variants influencing hsCRP levels in Indian population, this is not essentially a limitation of the study.

Epidemiological studies have consistently demonstrated that the risk of T2D and cardio-vascular disease (CVD) increase with higher hsCRP levels. In a study conducted on samples inclusive of those studied here, we earlier demonstrated that hsCRP levels were associated with T2D in a BMI independent manner [13]. In the light of hsCRP being a potential biomarker for CVD and T2D, the search for genetic variants influencing its expression is of great importance. With the realization that variant frequencies and haplotype structures vary considerably between ethnic groups, the present study provides a clear picture of genetic factors associated with plasma hsCRP levels in Indian population. Identification of genetic regulators of hsCRP levels might help in the development of prognostic markers for detecting individuals at risk for CVD and T2D.

In conclusion, we identified rs3093059 in *CRP* and rs1892534 in *LEPR* as the major *cis* and *tran*s-acting contributor respectively, to the variance in hsCRP levels in North Indian population. Still further studies investigating variants from *CRP* and its regulatory genes are warranted to explain the total genetic control of circulating hsCRP levels.

## Supporting Information

Figure S1 Pairwise linkage disequilibrium (LD) between the selected SNPs in the genes investigated in the study. Left panel shows the plots with D' values and right panel shows plot with r^2^values. LD plots for North Indians were drawn using the genotype data from the present study whereas LD plots for Europeans were made from genotype data from HapMap.
(DOC)

Figure S2 Association of individual associated SNPs and their haplotypes with hsCRP levels. A: *CRP* and B: *IL6*. The −log_10_ *P* values are plotted against the respective SNPs and haplotype combinations formed by that SNP and all the SNPs before. The trend lines of −log_10_ *P* values for individual SNPs and highest −log_10_ *P* values for haplotypes are drawn for the comparison of association of SNPs and haplotypes.
(DOC)

Table S1 Details of the polymorphisms selected for this study. MAF: Minor Allele Frequency; HWE: Hardy-Weinberg equilibrium. * MAF, Genotype distribution and HWE estimations have been done in non-diabetic subjects alone. ^†^ Tri-allelic variant. The two less-common alleles (T and A) were pooled. ^‡^ MAF<0.05. ^§^Assay failure. ^∥^ Significant deviation from HWE. Chromosome positions are relative to NCBI Build 36.3 assembly, dbSNP b126.
(DOC)

Table S2 Descriptive characteristics of the study populations. Data are presented as median values (inter-quartile ranges). N: Number of subjects; TC: total cholesterol; LDL-C: low-density lipoprotein cholesterol; HDL-C: high-density lipoprotein cholesterol; TG: triglyceride; hsCRP: high sensitivity C-reactive protein.
(DOC)

Table S3 Haplotype association with hsCRP levels. β: Beta. β and *P* values presented were adjusted for age, sex, BMI, and smoking/tobacco chewing for analysis done in non-diabetic subjects; age, sex, BMI, smoking/tobacco chewing, and medication for diabetic subjects; and age, sex, BMI, smoking/tobacco chewing, disease status, and medication for all subjects taken together. *P*_perm_: *P* value obtained after 10,000 permutations.
(DOC)
