# Supplementary material for: Common Variants in CRP and LEPR Influence High Sensitivity C-Reactive Protein Levels in North Indians
Source: PLoS One. 2011 Sep 8;6(9):e24645. doi: 10.1371/journal.pone.0024645 (PMC3169613; doi:10.1371/journal.pone.0024645)

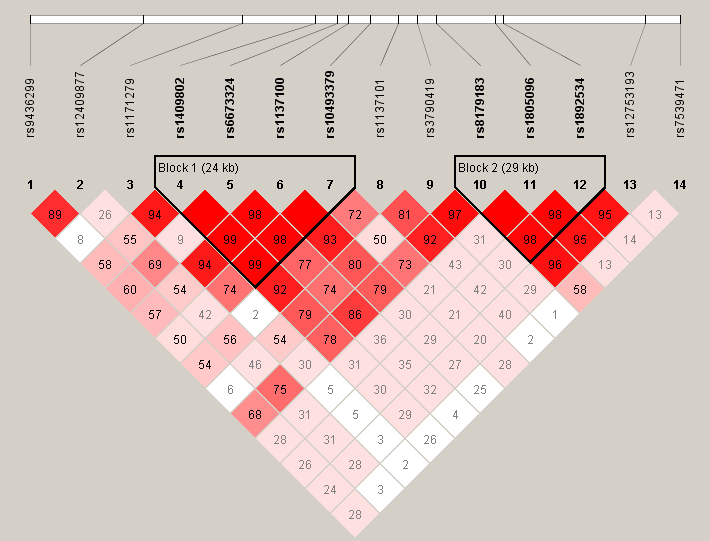

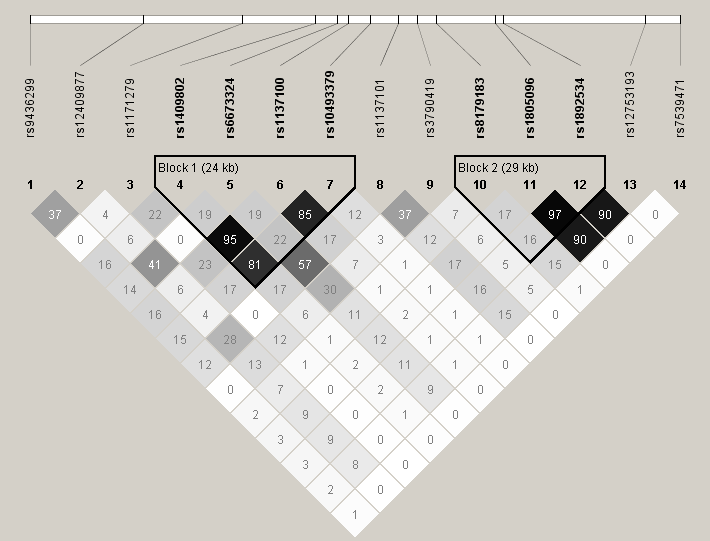


***LEPR***

North Indians

Europeans

North Indians


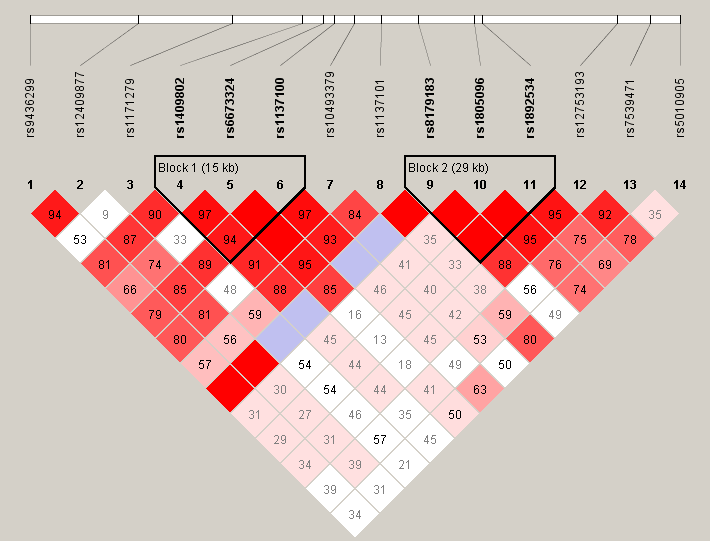

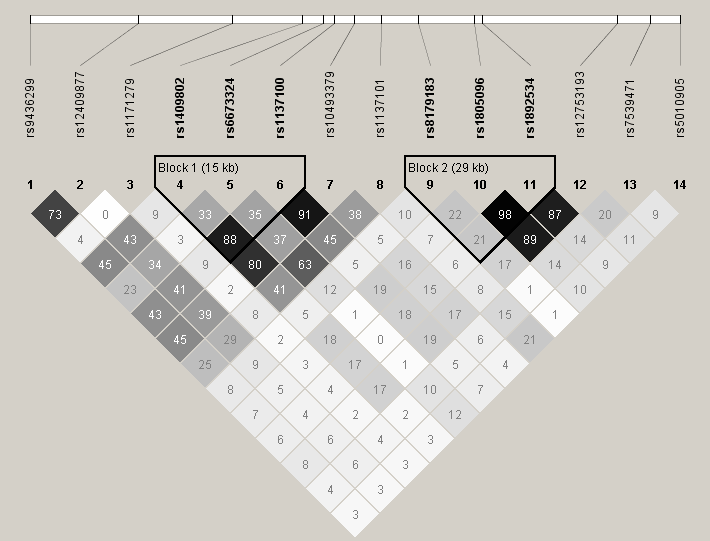


Europeans


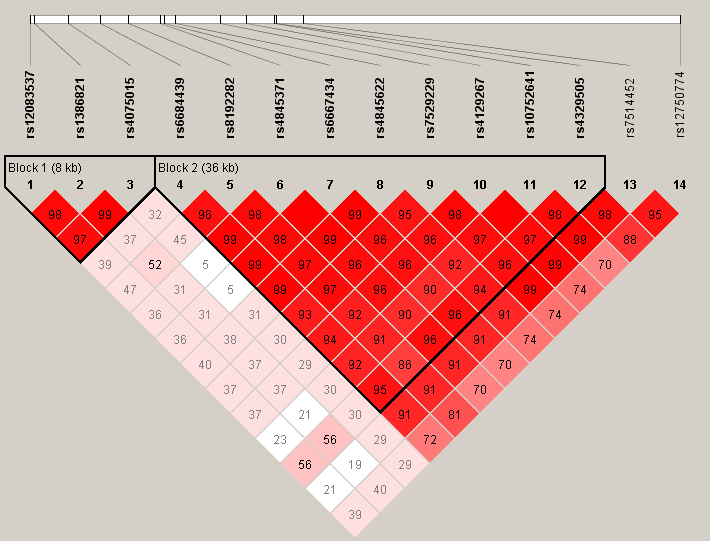

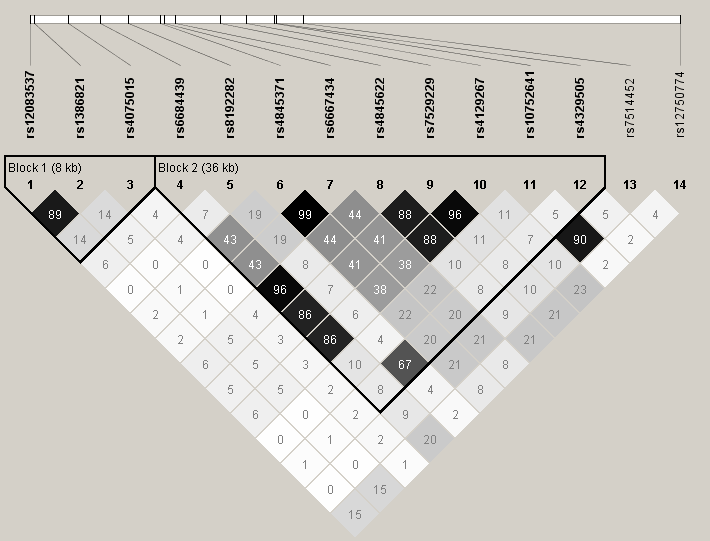


***IL6R***

North Indians

Europeans


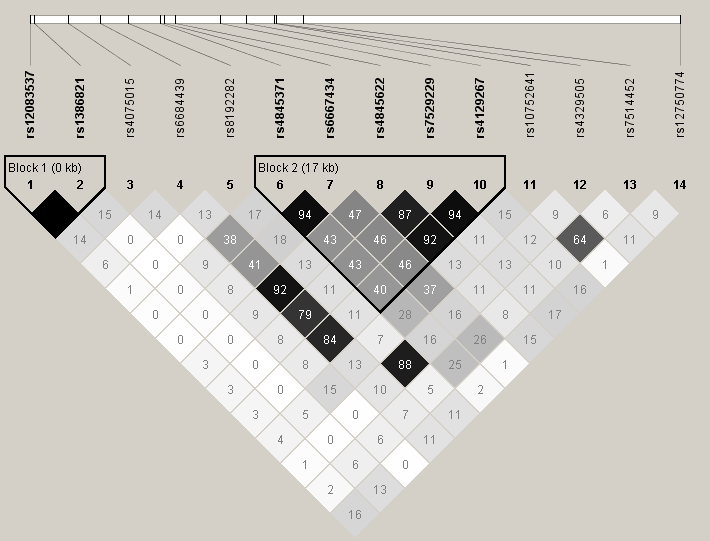

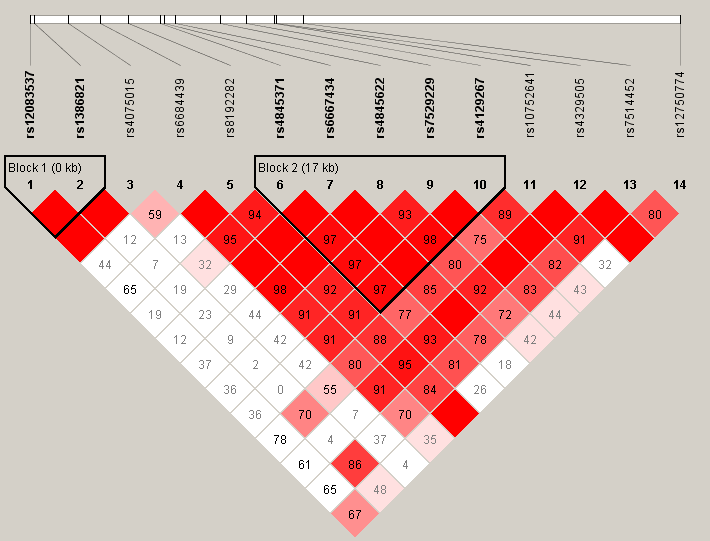


Europeans

***CRP***


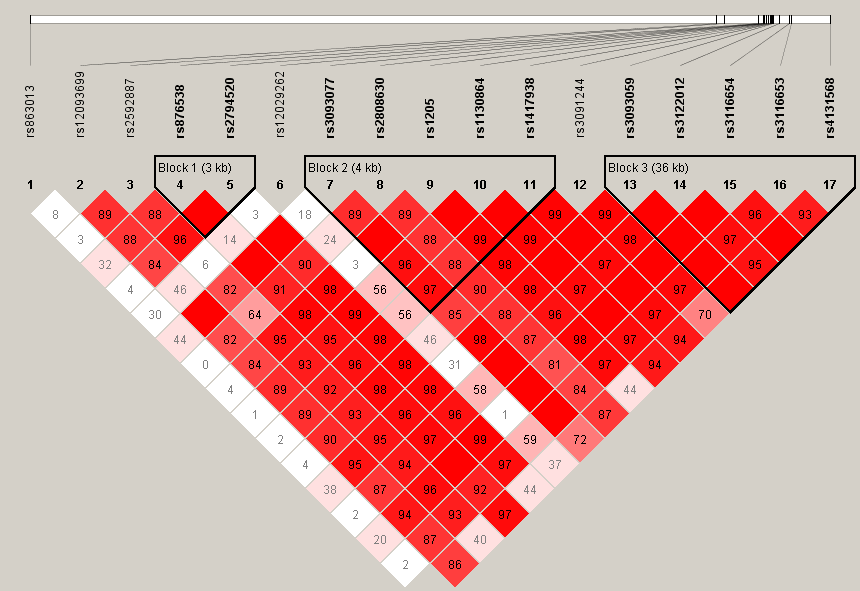

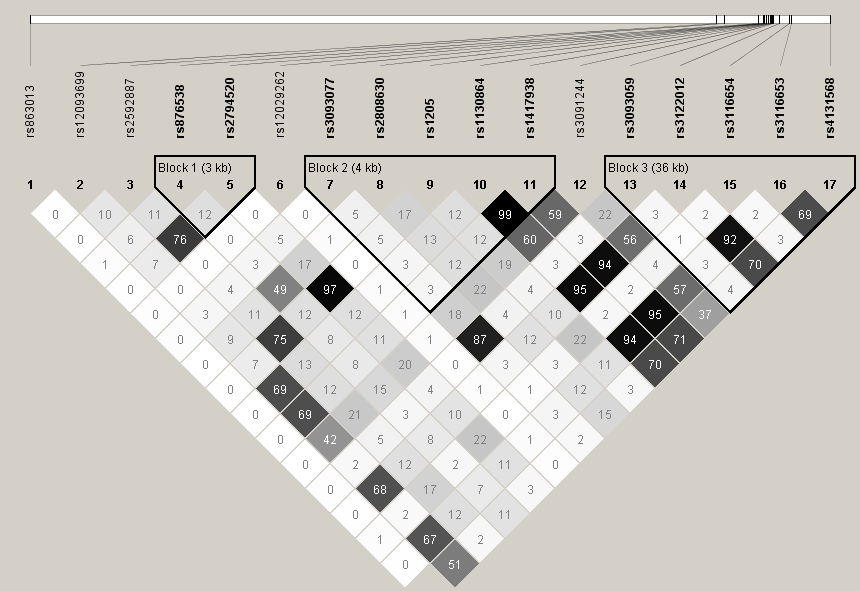

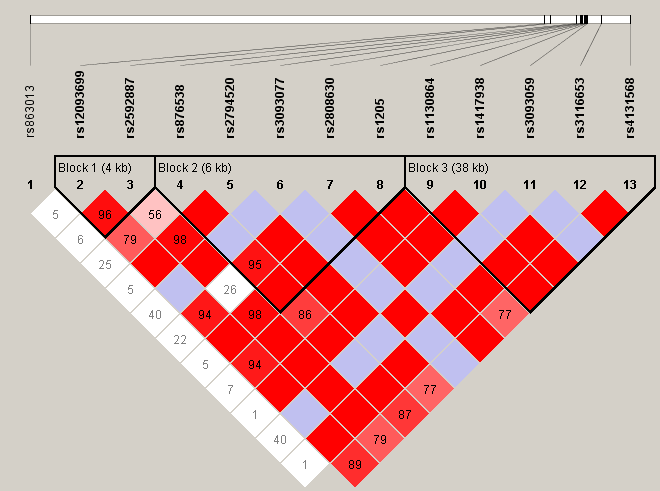

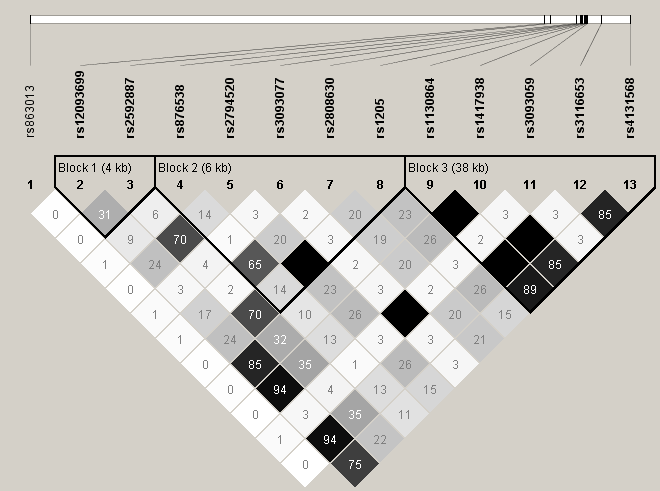


North Indians

Europeans


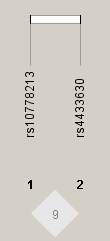


***GCKR***

**12q13.2**

North Indians

North Indians


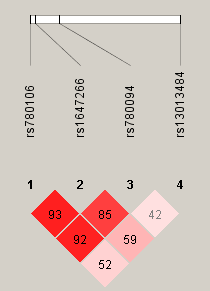

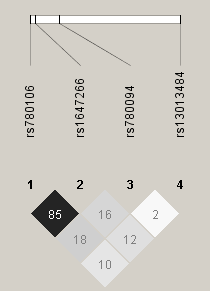

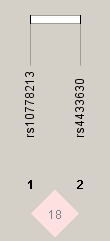


Europeans

Europeans


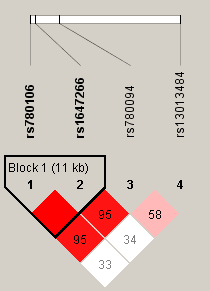

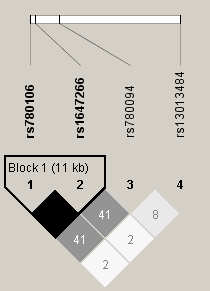

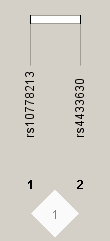

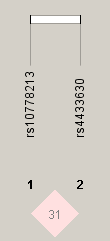


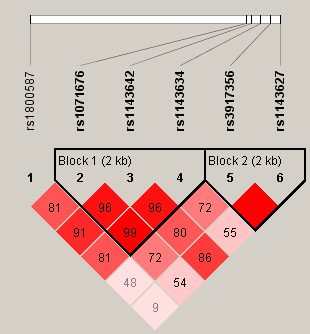

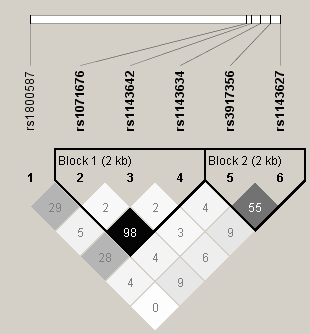


***IL1B***

North Indians

Europeans


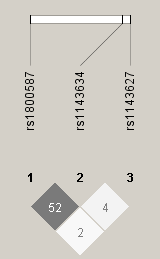

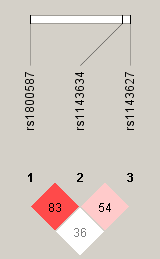


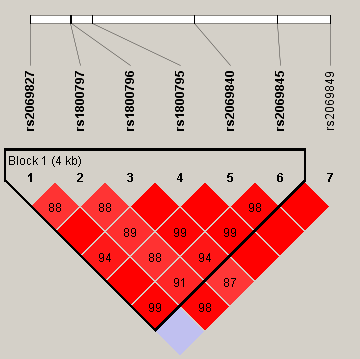

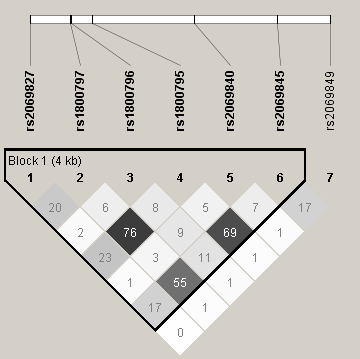

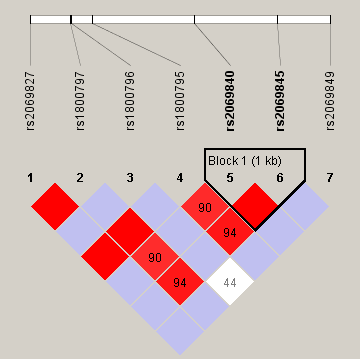

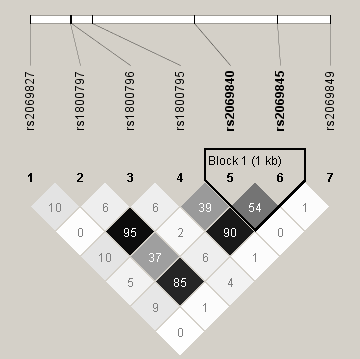


***IL6***

North Indians

Europeans


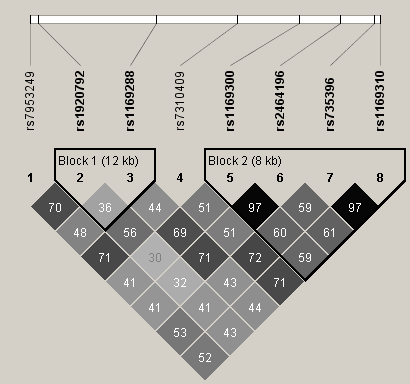

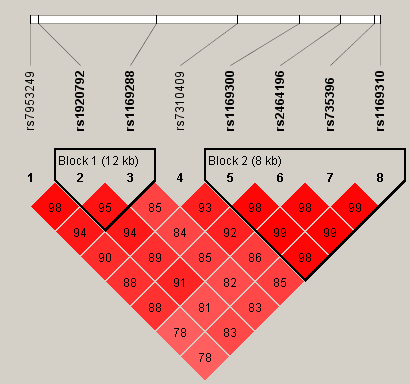


***HNF1A***

North Indians

Europeans


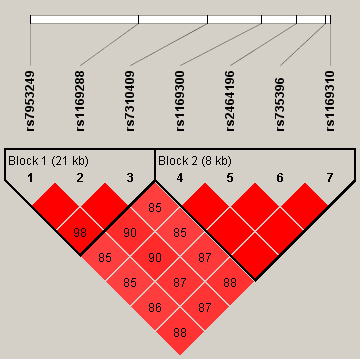

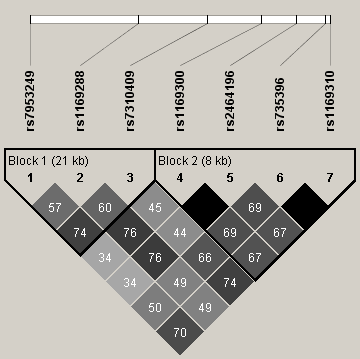


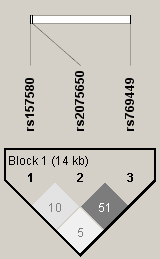

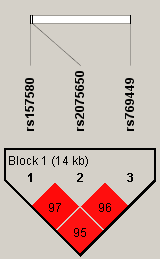


North Indians

***APOE***

Europeans


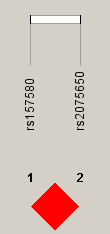

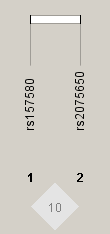

Supplement: Figure S1 — Pairwise linkage disequilibrium (LD) between the selected SNPs in the genes investigated in the study. Left panel shows the plots with D' values and right panel shows plot with r2values. LD plots for North Indians were drawn using the genotype data from the present study whereas LD plots for Europeans were made from genotype data from HapMap. (DOC) [file pone.0024645.s001.doc]
