# Supplementary figures and images for: Common Variants in CRP and LEPR Influence High Sensitivity C-Reactive Protein Levels in North Indians
Source: PLoS One. 2011 Sep 8;6(9):e24645. doi: 10.1371/journal.pone.0024645 (PMC3169613; doi:10.1371/journal.pone.0024645)

**Figure S2A**


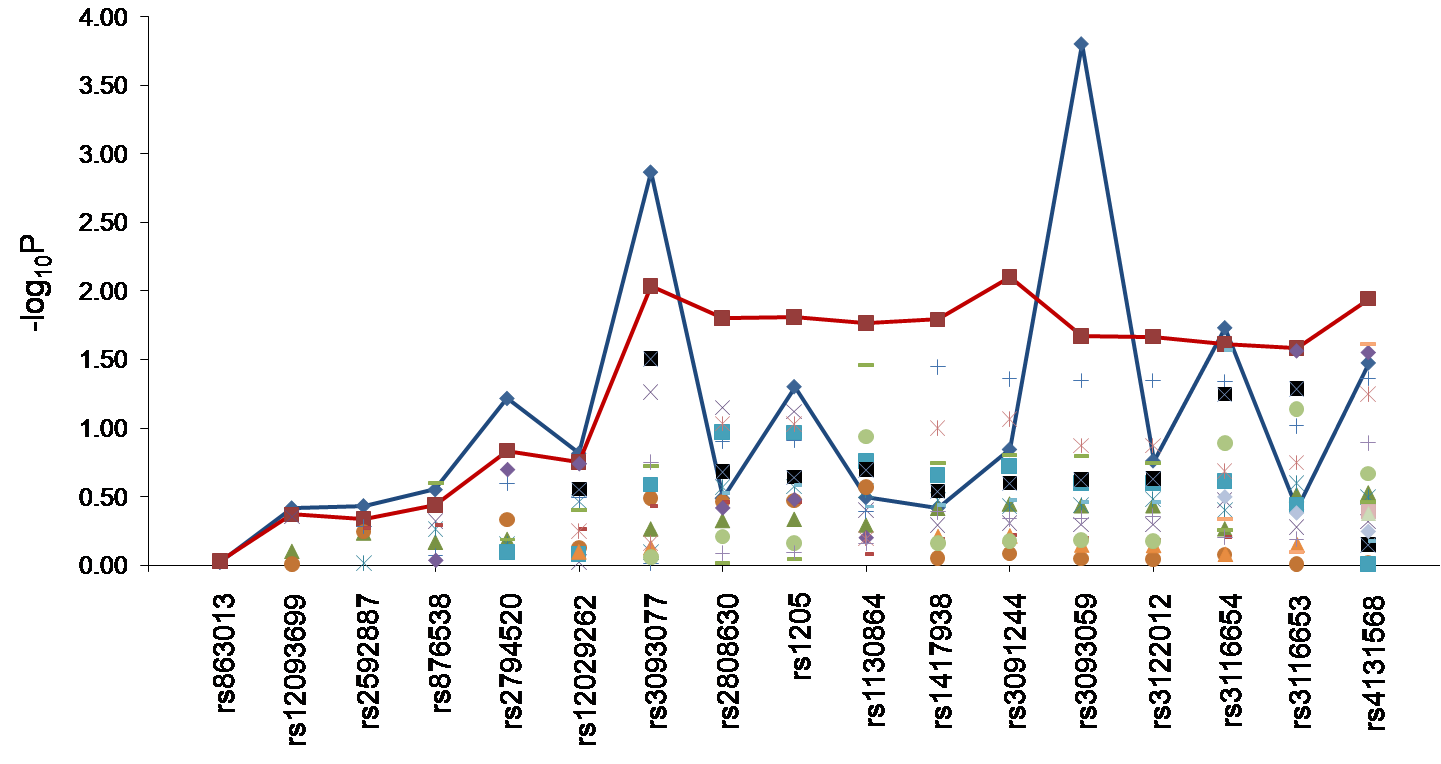

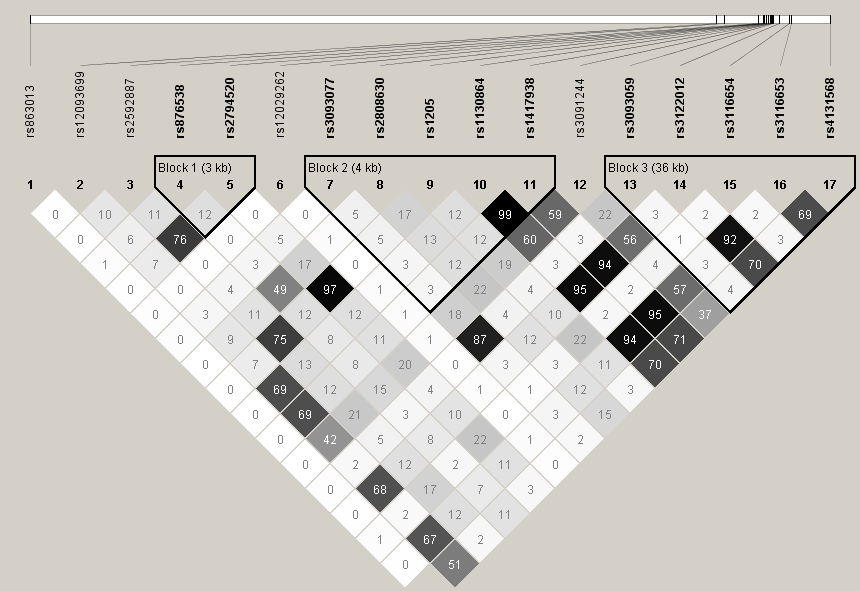


**Figure S2B**


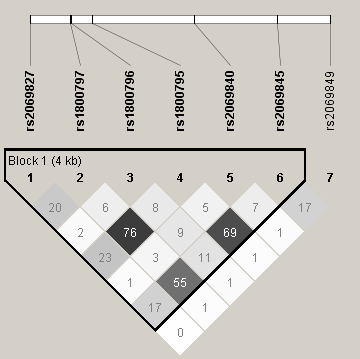

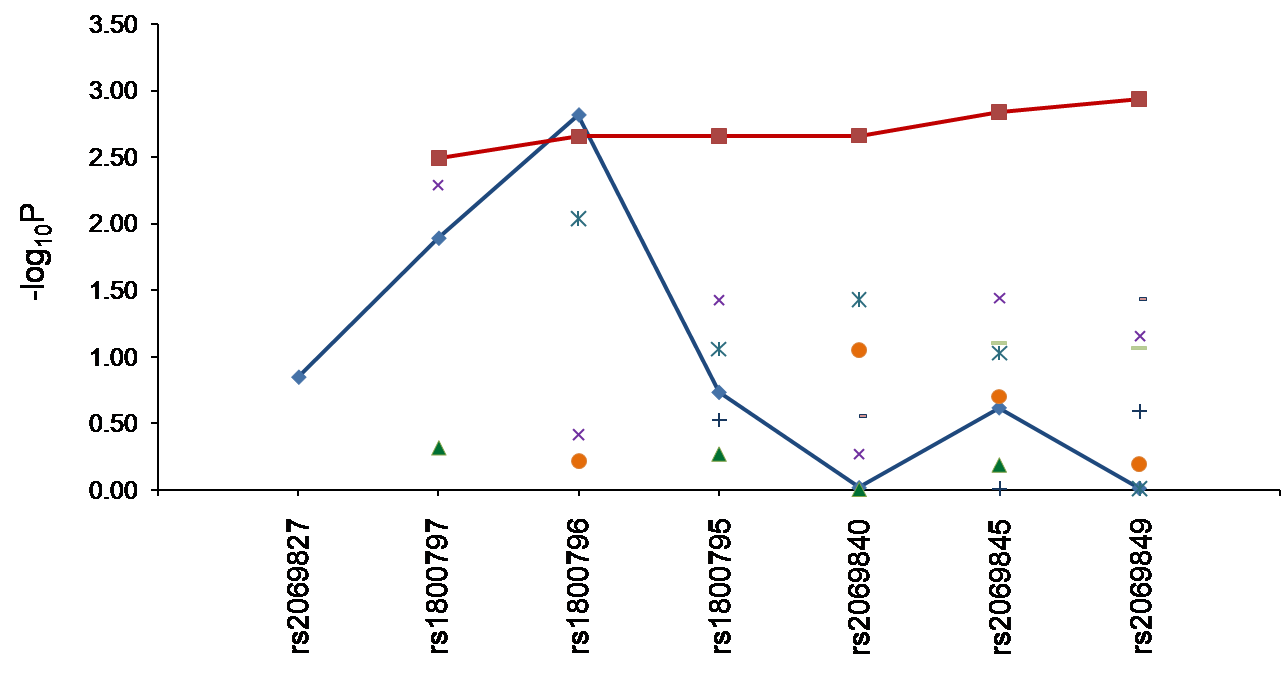

Supplement: Figure S2 — Association of individual associated SNPs and their haplotypes with hsCRP levels. A: CRP and B: IL6. The −log10 P values are plotted against the respective SNPs and haplotype combinations formed by that SNP and all the SNPs before. The trend lines of −log10 P values for individual SNPs and highest −log10 P values for haplotypes are drawn for the comparison of association of SNPs and haplotypes. (DOC) [file pone.0024645.s002.doc]
