# Supplementary material for: Common Variants in CRP and LEPR Influence High Sensitivity C-Reactive Protein Levels in North Indians
Source: PLoS One. 2011 Sep 8;6(9):e24645. doi: 10.1371/journal.pone.0024645 (PMC3169613; doi:10.1371/journal.pone.0024645)
Supplement: Table S1 — Details of the polymorphisms selected for this study. MAF: Minor Allele Frequency; HWE: Hardy-Weinberg equilibrium. * MAF, Genotype distribution and HWE estimations have been done in non-diabetic subjects alone. † Tri-allelic variant. The two less-common alleles (T and A) were pooled. ‡ MAF<0.05. §Assay failure. ∥ Significant deviation from HWE. Chromosome positions are relative to NCBI Build 36.3 assembly, dbSNP b126. (DOC) [file pone.0024645.s003.doc]

Table S1: Details of the polymorphisms selected for this study

| **Gene (Chromosome)** | **Polymorphism** | **Chromosomal location** | **Minor allele** | **Major allele** | **MAF*** | **Genotype distribution*** | **HWE*** |
| --- | --- | --- | --- | --- | --- | --- | --- |
| *LEPR* (1) | rs9436299 | 65665476 | G | T | 0.28 | 86/398/522 | 0.44 |
|  | rs12409877 | 65716460 | A | G | 0.46 | 214/471/289 | 0.40 |
|  | rs1171279 | 65761081 | G | A | 0.42 | 184/484/338 | 0.65 |
|  | rs1409802 | 65793939 | T | C | 0.15 | 33/242/731 | 0.03 |
|  | rs6673324 | 65803651 | A | G | 0.49 | 248/496/261 | 0.70 |
|  | rs1137100 | 65809029 | G | A | 0.16 | 38/247/720 | 0.007 |
|  | rs10493379 | 65818515 | T | C | 0.18 | 44/277/685 | 0.03 |
|  | rs1137101 | 65831101 | C | T | 0.49 | 241/500/264 | 0.90 |
|  | rs3790419 | 65839697 | G | A | 0.35 | 137/431/435 | 0.07 |
|  | rs13306522 ^‡^ | 65839973 | T | C | 0.03 | 0/53/953 | 1.00 |
|  | rs8179183 | 65848540 | G | C | 0.13 | 21/223/762 | 0.33 |
|  | rs1805096 | 65874845 | A | G | 0.48 | 225/521/260 | 0.26 |
|  | rs1892534 | 65878532 | A | G | 0.48 | 210/512/247 | 0.08 |
|  | rs12753193 | 65942267 | G | A | 0.48 | 213/503/257 | 0.28 |
|  | rs7539471 | 65957991 | A | G | 0.25 | 56/379/540 | 0.35 |
|  | rs5010905 | 65971811 | C | T | 0.24 | 36/216/359 | 0.65 |
| *IL6R* (1) | rs4845617 ^§^ | 152644522 |  |  |  |  |  |
|  | rs12083537 | 152647727 | G | A | 0.16 | 16/281/676 | 0.03 |
|  | rs1386821 | 152648673 | G | T | 0.15 | 16/270/719 | 0.11 |
|  | rs4075015 | 152655820 | T | A | 0.47 | 206/524/275 | 0.14 |
|  | rs4601580 ^§^ | 152661041 |  |  |  |  |  |
|  | rs6684439 | 152662463 | T | C | 0.31 | 90/412/456 | 0.88 |
|  | rs8192282 | 152668303 | T | C | 0.16 | 25/265/716 | 0.91 |
|  | rs4845371 | 152674964 | G | A | 0.49 | 243/513/250 | 0.57 |
|  | rs6667434 | 152675724 | G | A | 0.49 | 243/513/250 | 0.57 |
|  | rs4845622 | 152678043 | C | A | 0.32 | 100/399/434 | 0.55 |
|  | rs7529229 | 152687402 | G | A | 0.31 | 95/434/477 | 0.83 |
|  | rs4129267 | 152692888 | T | C | 0.29 | 65/421/461 | 0.02 |
|  | rs8192284 | 152693594 | G | T | 0.31 | 61/353/350 | 0.03 |
|  | rs2228146 ^§^ | 152693674 |  |  |  |  |  |
|  | rs10752641 | 152698666 | C | G | 0.21 | 44/330/632 | 0.92 |
|  | rs4329505 | 152699044 | C | T | 0.16 | 30/238/675 | 0.11 |
|  | rs7514452 | 152704708 | C | T | 0.19 | 40/312/654 | 0.69 |
|  | rs12750774 | 152783101 | A | G | 0.16 | 23/264/695 | 0.81 |
| *CRP* (1) | rs863013 | 157466644 | T | G | 0.27 | 82/366/531 | 0.11 |
|  | rs12093699 | 157914612 | A | G | 0.2 | 39/311/627 | 0.92 |
|  | rs2592887 | 157919563 | A | G | 0.33 | 114/423/437 | 0.47 |
|  | rs876538 | 157942341 | A | G | 0.22 | 50/304/566 | 0.29 |
|  | rs2794520 | 157945440 | T | C | 0.29 | 91/390/497 | 0.25 |
|  | rs12029262 | 157945820 | C | G | 0.11 | 14/178/776 | 0.31 |
|  | rs3093077 | 157946260 | G | T | 0.12 | 20/204/756 | 0.18 |
|  | rs2808630 | 157947492 | C | T | 0.35 | 115/438/404 | 0.89 |
|  | rs1205 | 157948857 | A | G | 0.29 | 90/407/509 | 0.49 |
|  | rs1130864 | 157949715 | A | G | 0.24 | 51/343/536 | 0.72 |
|  | rs1800947 ^‡^ | 157950062 | C | G | 0.02 | 0/43/963 | 1.00 |
|  | rs1417938 | 157950810 | T | A | 0.23 | 51/355/598 | 0.93 |
|  | rs3091244 ^†^ | 157951289 | T/A | C | 0.34 | 128/412/437 | 0.05 |
|  | rs3093059 | 157951760 | C | T | 0.12 | 18/188/766 | 0.11 |
|  | rs3122012 | 157955947 | C | T | 0.22 | 51/306/586 | 0.18 |
|  | rs3116654 | 157962385 | C | T | 0.09 | 8/164/788 | 1.00 |
|  | rs3116653 | 157963534 | G | C | 0.22 | 49/341/585 | 1.00 |
|  | rs4131568 | 157988680 | A | G | 0.27 | 75/352/518 | 0.18 |
| *GCKR* (2) | rs780106 | 27535102 | C | A | 0.42 | 170/489/323 | 0.56 |
|  | rs1647266 | 27546989 | C | T | 0.42 | 168/490/323 | 0.47 |
|  | rs780094 | 27594741 | T | C | 0.24 | 61/353/591 | 0.38 |
|  | rs13013484 | 27842325 | G | A | 0.33 | 111/417/451 | 0.34 |
| *IL1A* (2) | rs1800587 | 113259431 | T | C | 0.33 | 105/430/440 | 1.0 |
| *IL1B* (2) | rs1071676 | 113303904 | C | G | 0.18 | 30/299/676 | 0.75 |
|  | rs1143642 | 113305024 | T | C | 0.11 | 14/196/769 | 0.64 |
|  | rs1143634 | 113306861 | A | G | 0.18 | 30/296/679 | 0.83 |
|  | rs3917356 | 113308834 | A | G | 0.28 | 83/398/523 | 0.58 |
|  | rs1143627 | 113310858 | A | G | 0.42 | 182/478/346 | 0.47 |
|  | rs16944 ^§^ | 113311338 |  |  |  |  |  |
| *SLC1A3* (5) | rs7730843 || | 36578069 | A | G | 0.12 | 25/139/657 | 1.6×10^-5^ |
| *IL6* (7) | rs2069827 | 22731981 | T | G | 0.05 | 4/101/901 | 0.53 |
|  | rs1800797 | 22732746 | A | G | 0.17 | 26/289/662 | 0.44 |
|  | rs1800796 | 22732771 | G | C | 0.28 | 82/397/527 | 0.58 |
|  | rs1800795 | 22733170 | G | C | 0.18 | 31/289/680 | 1.00 |
|  | rs3087226 ^‡^ | 22733374 | C | G | 0 |  |  |
|  | rs2069840 | 22735097 | G | C | 0.21 | 45/324/637 | 0.63 |
|  | rs2069845 | 22736674 | G | A | 0.24 | 61/355/589 | 0.43 |
|  | rs2069849 | 22737681 | A | G | 0.05 | 3/104/899 | 1.00 |
|  | rs2069861 ^‡^ | 22738179 | A | G | 0.03 | 0/57/949 | 1.00 |
| *FAM13C* (10) | rs6481464 | 60790490 | T | C | 0.29 | 85/371/482 | 0.27 |
| 12q23.2 (12) | rs10778213 | 102019281 | C | T | 0.49 | 240/454/260 | 0.14 |
|  | rs4433630 | 102174161 | T | C | 0.31 | 102/327/421 | 0.002 |
| *HNF1A* (12) | rs7953249 | 119888107 | A | G | 0.44 | 198/435/321 | 0.03 |
|  | rs1920792 | 119888967 | G | A | 0.36 | 148/435/422 | 0.04 |
|  | rs1169288 | 119901033 | G | T | 0.41 | 177/469/360 | 0.27 |
|  | rs1800574 ^§^ | 119901247 |  |  |  |  |  |
|  | rs7310409 | 119909244 | G | A | 0.47 | 214/485/280 | 0.89 |
|  | rs1169300 | 119915608 | A | G | 0.41 | 165/459/347 | 0.55 |
|  | rs2071190 || | 119915655 | T | A | 0.13 | 0/260/711 | 2.7×10^-9^ |
|  | rs1169302 ^§^ | 119916685 |  |  |  |  |  |
|  | rs2464196 | 119919810 | T | C | 0.4 | 168/476/361 | 0.60 |
|  | rs735396 | 119923227 | A | G | 0.48 | 237/458/281 | 0.06 |
|  | rs1169310 | 119923816 | C | T | 0.48 | 246/475/285 | 0.09 |
| *APOE* (19) | rs157580 | 50087106 | G | A | 0.46 | 206/442/278 | 0.23 |
|  | rs2075650 | 50087459 | G | A | 0.11 | 11/192/772 | 1.00 |
|  | rs769449 | 50101842 | A | G | 0.07 | 4/121/855 | 1.00 |
|  | rs4420638 || | 50114786 | C | T | 0.1 | 0/206/784 | 9.8×10^-6^ |

MAF: Minor Allele Frequency; HWE: Hardy-Weinberg equilibrium

* MAF, Genotype distribution and HWE estimations have been done in non-diabetic subjects alone

^†^ Tri-allelic variant. The two less-common alleles (T and A) were pooled

^‡^ MAF<0.05

^§^Assay failure

^||^ Significant deviation from HWE

Chromosome positions are relative to NCBI Build 36.3 assembly, dbSNP b126
