# Supplementary material for: Common Variants in CRP and LEPR Influence High Sensitivity C-Reactive Protein Levels in North Indians
Source: PLoS One. 2011 Sep 8;6(9):e24645. doi: 10.1371/journal.pone.0024645 (PMC3169613; doi:10.1371/journal.pone.0024645)
Supplement: Table S2 — Descriptive characteristics of the study populations. Data are presented as median values (inter-quartile ranges). N: Number of subjects; TC: total cholesterol; LDL-C: low-density lipoprotein cholesterol; HDL-C: high-density lipoprotein cholesterol; TG: triglyceride; hsCRP: high sensitivity C-reactive protein. (DOC) [file pone.0024645.s004.doc]

**Table S2: Descriptive characteristics of the study populations**

| **Characteristics** | **Initial phase** |  | **Follow up** |  |
| --- | --- | --- | --- | --- |
|  | **Patients with type 2 diabetes** | **Non-diabetic subjects** | **Patients with type 2 diabetes** | **Non-diabetic subjects** |
| N | 1019 | 1006 | 1047 | 1038 |
| Male/Female (%) | 58/42 | 60/40 | 59/41 | 50/50 |
| Age (years) | 53 (45-62) | 50 (44-60) | 55 (49-62) | 54 (45-64) |
| BMI (kg/m2) | 24.8 (22.5-27.5) | 23.8 (20.4-26.9) | 25.9 (23.2-29.3) | 25.4 (22.7-28.5) |
| TC (mmol/L) | 4.20 (3.50-5.00) | 4.40 (3.7-5.10) | 4.64 (3.86-5.42) | 4.91 (4.22-5.52) |
| LDL-C (mmol/L) | 2.57 (1.99-3.36) | 2.79 (2.33-3.41) | 2.73 (2.10-3.42) | 3.01 (2.49-3.51) |
| HDL-C (mmol/L) | 1.03 (0.88-1.22) | 1.06 (0.88-1.28) | 1.11 (0.94-1.34) | 1.24 (1.06-1.46) |
| TG (mmol/L) | 1.60 (1.10-2.20) | 1.30 (1.00-1.80) | 1.43 (0.98-2.13) | 1.22 (0.86-1.64) |
| hsCRP (µg/mL) | 2.2 (0.9-4.7) | 1.3 (0.6-3.0) | 1.9 (0.9-3.4) | 1.6 (0.9-3.0) |

Data are presented as median values (inter-quartile ranges)

N: Number of subjects; TC: total cholesterol; LDL-C: low-density lipoprotein cholesterol; HDL-C: high-density lipoprotein cholesterol; TG: triglyceride;

hsCRP: high sensitivity C-reactive protein
