# Supplementary material for: Common Variants in CRP and LEPR Influence High Sensitivity C-Reactive Protein Levels in North Indians
Source: PLoS One. 2011 Sep 8;6(9):e24645. doi: 10.1371/journal.pone.0024645 (PMC3169613; doi:10.1371/journal.pone.0024645)
Supplement: Table S3 — Haplotype association with hsCRP levels. β: Beta. β and P values presented were adjusted for age, sex, BMI, and smoking/tobacco chewing for analysis done in non-diabetic subjects; age, sex, BMI, smoking/tobacco chewing, and medication for diabetic subjects; and age, sex, BMI, smoking/tobacco chewing, disease status, and medication for all subjects taken together. P perm: P value obtained after 10,000 permutations. (DOC) [file pone.0024645.s005.doc]

**Table S3: Haplotype association with hsCRP levels**

| **Subjects** | **Gene** | **Haplotype** | **Polymorphisms** | **Frequency** | **β** | ***P*** | ***P*perm** |
| --- | --- | --- | --- | --- | --- | --- | --- |
| Non-diabetic | *CRP* | GATCTTCG | rs1130864/rs1417938/ rs3091244/rs3093059/ rs3122012/rs3116654/ rs316653/rs4131568 | 0.11 | 0.32 | 1.4×10^-4^ | 9.0×10^-4^ |
| T2D | *CRP* | GATCTTCG | rs1130864/rs1417938/ rs3091244/rs3093059/ rs3122012/rs3116654/ rs316653/rs4131568 | 0.1 | 0.3 | 4.6×10^-3^ | 0.03 |
| All | *CRP* | GATCTTCG | rs1130864/rs1417938/ rs3091244/rs3093059/ rs3122012/rs3116654/ rs316653/ rs4131568 | 0.1 | 0.32 | 1.8×10^-6^ | 1.0×10^-4^ |
|  | *CRP* | GG | rs1205/rs1130864 | 0.47 | 0.16 | 3.3×10^-4^ | 1.3×10^-3^ |
|  | *CRP* | GT | rs876538/rs2794520 | 0.3 | -0.16 | 4.8×10^-4^ | 1.8×10^-3^ |
|  | *CRP* | AG | rs1205/rs1130864 | 0.29 | -0.16 | 8.8×10^-4^ | 2.6×10^-3^ |
|  | *IL6* | GGGCCA | rs2069827/rs1800797/ rs1800796/rs1800795/ rs2069840/rs2069845 | 0.28 | -0.16 | 3.9×10^-4^ | 3.0×10^-3^ |

β: Beta

β* *and *P* values presented were adjusted for age, sex, BMI, and smoking/tobacco chewing for analysis done in non-diabetic subjects; age, sex, BMI, smoking/tobacco chewing, and medication for diabetic subjects; and age, sex, BMI, smoking/tobacco chewing, disease status, and medication for all subjects taken together

*P*_perm_: *P* value obtained after 10,000 permutations
